# Delaying cardiac aging: potential mechanisms centered on PANoptosis and targeted intervention strategies

**DOI:** 10.3389/fcvm.2026.1759908

**Published:** 2026-02-24

**Authors:** Yuhe Shu, Shan Li, Shuyu Yang, Simin Zhang, Bo Li, Li Dong

**Affiliations:** 1 The Affiliated Traditional Chinese Medicine Hospital, Southwest Medical University, Luzhou, China; 2 The Key Laboratory of Integrated Traditional Chinese and Western Medicine for Prevention and Treatment of Digestive System Diseases of Luzhou City, The Affiliated Traditional Chinese Medicine Hospital, Southwest Medical University, Luzhou, China

**Keywords:** cardiac aging, myocardial dysfunction, PANoptosis, programmed cell death, targeted intervention

## Abstract

As the vital power organ of the human body, the health of the heart directly determines an individual's quality of life and longevity. With the accelerating global aging population, cardiac aging-related diseases have become a major public health threat. Although existing interventions (e.g., senolytics) can delay cardiac aging to some extent, their efficacy remains limited, necessitating the exploration of novel mechanisms to develop more effective therapeutic strategies. In recent years, PANoptosis—an integrated cell death pathway—has emerged as a new research focus in cardiac aging. It may contribute to cardiac functional decline by accelerating cardiomyocyte loss, fibrosis, and chronic inflammation. Targeting PANoptosis-based intervention strategies (e.g., gene editing, RNAi, combination therapy, and novel delivery systems) has demonstrated significant therapeutic potential, offering new preclinical avenues to delay or alleviate cardiac aging. This review summarizes the molecular mechanisms and roles of PANoptosis in cardiac aging, including its regulatory networks, key evidence driving cardiac aging, and targeted intervention strategies, thereby providing a theoretical foundation for developing PANoptosis-targeted therapies against cardiac aging.

## 1 Introduction

The heart, as the vital pump of the human body, plays a decisive role in determining an individual's quality of life and lifespan. With the accelerating trend of global population aging, the incidence of cardiac aging-related diseases—such as heart failure and arrhythmias—has been rising annually, emerging as a critical public health challenge (1). Cardiac aging represents a complex pathophysiological process characterized by progressive, degenerative changes in cardiac structure and function over time, ultimately leading to declining cardiac output that fails to meet metabolic demands (2). Although significant progress has been made in managing acute cardiovascular events worldwide, therapeutic strategies to delay cardiac aging remain limited. Current interventions (e.g., senolytics like dasatinib and quercetin) primarily target senescent cell clearance but demonstrate restricted efficacy in restoring cardiac function (3). This underscores an urgent need to elucidate novel mechanisms driving cardiac aging.

Programmed cell death (PCD) exhibits a dual role in cardiac homeostasis. While apoptosis has long been implicated in age-related cardiomyocyte loss (4), its inhibition in aging models fails to fully restore cardiac function (5). Emerging evidence suggests that other inflammatory PCD pathways—pyroptosis and necroptosis—are concurrently activated during cardiac aging, releasing proinflammatory cytokines (e.g., IL-1β, IL-18) to exacerbate tissue damage (6, 7). However, conventional research has focused narrowly on isolated death pathways, overlooking their crosstalk. This approach not only inadequately explains the complex pathology of cardiac aging but also limits our understanding of how integrated cell death networks regulate this process.

PANoptosis, a recently defined lytic cell death modality, integrates core molecular mechanisms of pyroptosis, apoptosis, and necroptosis into a dynamically regulated “death signaling network” (8). As a unique PCD paradigm, it transcends classical boundaries of these pathways by forming the PANoptosome complex, which orchestrates caspase family members (e.g., caspase-1/3/8), inflammasomes (e.g., NLRP3), and RIP kinases (e.g., RIPK3) (9). PANoptosis has demonstrated distinctive roles across physiological and pathological contexts. For instance, studies have uncovered its involvement in inflammatory responses, tumorigenesis, and infectious diseases (10, 11). Despite these advances, its contribution to organ-specific aging—particularly in cardiac aging, a pivotal biological process—remains poorly explored.

Thus, investigating PANoptosis in cardiac aging not only offers novel insights into its pathophysiology but also presents opportunities to develop targeted interventions. This review systematically deciphers the molecular architecture of PANoptosis and its activation mechanisms in cardiac aging, synthesizes preclinical and clinical evidence linking PANoptosis to aging-related cardiac phenotypes, and proposes therapeutic strategies targeting PANoptosis to mitigate age-dependent cardiac decline. By bridging cell death biology and geriatric cardiology, we highlight PANoptosis as a transformative target for anti-aging therapeutics.

## 2 Characteristics and mechanisms of cardiac aging

As a pivotal component of organismal aging, cardiac aging manifests multifaceted features driven by intricate molecular and cellular mechanisms that collectively impair cardiac function through progressive deterioration (12).

### 2.1 Macroscopic features: structural remodeling and functional decline

At the macroscopic level, structural remodeling and functional deterioration represent hallmark manifestations of cardiac aging. Morphologically, myocardial hypertrophy emerges as a compensatory response to chronic hemodynamic stress (e.g., hypertension or valvular diseases) (13). While initially adaptive, sustained hypertrophy disrupts myocardial energetics and accelerates cellular senescence (14). Concurrently, TGF-β signaling dysregulation promotes age-dependent myocardial fibrosis, where extracellular matrix deposition increases myocardial stiffness and impairs diastolic function (15). Valvular calcification exacerbates functional impairment by altering hemodynamics via stenosis or regurgitation (16). Coronary atherosclerosis reduces myocardial perfusion through plaque formation and endothelial dysfunction, elevating ischemic risk (17). Age-related degeneration of the cardiac conduction system predisposes to arrhythmias by altering ion channel expression and gap junction connectivity (18, 19).

Functional decline presents as a dual impairment: reduced peak ejection fraction and diminished early diastolic filling rate collectively characterize “biventricular failure” (20). Systolic dysfunction stems from decreased myofilament calcium sensitivity and impaired excitation-contraction coupling, reducing stroke volume (21, 22). Diastolic dysfunction arises from increased myocardial stiffness and delayed relaxation, elevating ventricular filling pressures (23, 24). Histological analyses reveal that interstitial fibrosis (collagen deposition) significantly increases ventricular stiffness, while coronary calcification progresses with aging—collectively leading to a marked reduction in diastolic filling rates (25). Autonomic dysfunction, marked by reduced heart rate variability, reflects diminished vagal tone and heightened sympathetic activity, promoting arrhythmias and decompensation (26, 27).

### 2.2 Cellular and molecular mechanisms

Mitochondrial dysfunction constitutes a central mechanism. As the cellular powerhouses, mitochondria exhibit functional decline as a key hallmark of cardiac aging (28). Age-related reductions in mitochondrial biogenesis and dynamics impair ATP production, while elevated reactive oxygen species (ROS) generation induces macromolecular oxidative damage (29). Mitophagy—a critical process for clearing damaged mitochondria—becomes less efficient with aging, leading to accumulated dysfunctional mitochondria that exacerbate cardiac impairment (30, 31).

**Figure 1:**
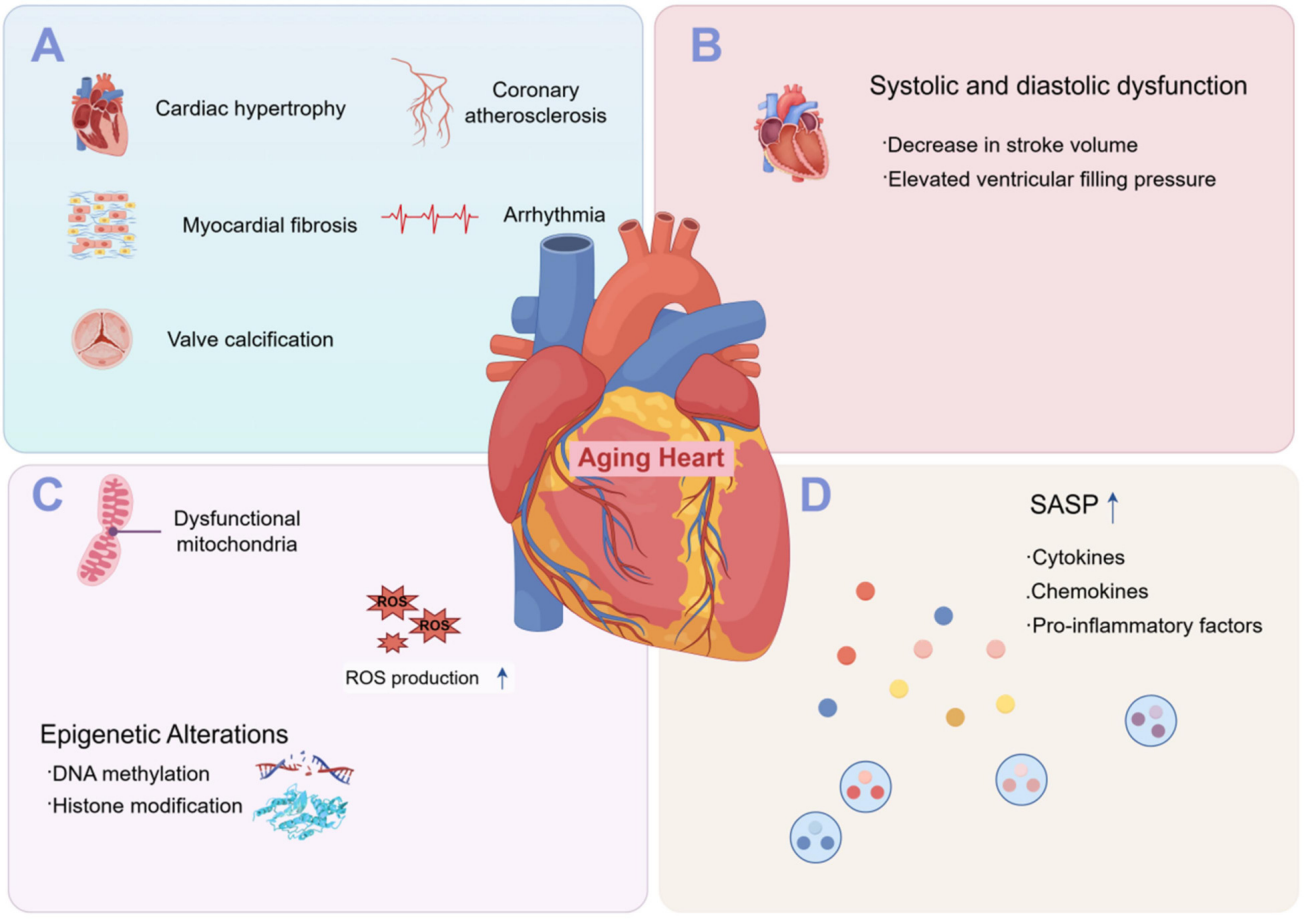
Pathophysiological features of cardiac aging at structural, functional, and molecular levels. **(A)** Key structural alterations: Cardiac hypertrophy, coronary atherosclerosis, myocardial fibrosis, arrhythmia, valve calcification. **(B)** Systolic/diastolic dysfunction: Reduced myofilament calcium sensitivity impairs systole, fibrosis-induced stiffness impairs diastole, elevating filling pressures. **(C)** Molecular changes: Mitochondrial dysfunction, epigenetic alterations. **(D)** Chronic inflammation: SASP from senescent cells releases proinflammatory factors, activating immune cells; inflammasome-mediated IL-1β/IL-18 amplifies inflammation, exacerbating damage.This figure was drawn by Figdraw.

Telomere attrition triggers DNA damage responses that induce cellular senescence, limiting cardiomyocyte regenerative capacity (32, 33). Epigenetic alterations (e.g., histone modifications and DNA methylation) regulate age-dependent transcriptional changes, disrupting metabolic pathways and extracellular matrix homeostasis (34). Chronic inflammation represents another critical feature (35). The senescence-associated secretory phenotype (SASP)—a major inflammatory driver—releases proinflammatory cytokines, chemokines, and proteases (36). These factors activate immune cells, exacerbating tissue damage and fibrosis. Inflammasomes, as key inflammatory regulators, promote IL-1β and IL-18 release upon activation (37). These interdependent processes collectively form a complex network driving cardiac aging (Figure 1).

## 3 Molecular mechanisms and regulatory network of PANoptosis

The concept of PANoptosis was first proposed in 2019 by a research team from St. Jude Children's Research Hospital in the United States. During studies on cell death mechanisms, researchers discovered that under certain conditions, cell death does not occur via a single pathway (apoptosis, necrosis, or pyroptosis) but rather involves a complex process integrating multiple death pathways. This novel form of programmed cell death was named PANoptosis, and it was identified as being mediated by a multiprotein complex termed the PANoptosome (38). Its core pathways can be divided into three stages: damage signal sensing, PANoptosome complex assembly, and downstream effector execution.

### 3.1 Sensing of upstream damage signals

In the aging myocardial microenvironment, mitochondrial dysfunction triggers critical endogenous danger signals. Reduced mitophagic efficiency leads to mtDNA leakage into the cytoplasm, activating innate immune responses via the cGAS-STING pathway and the absent in melanoma 2 (AIM2) inflammasome (39, 40). The N-terminus of Z-DNA binding protein 1 (ZBP1) contains two Z-type nucleic acid-binding domains (ZBDs), Zα1 and Zα2, which specifically recognize left-handed Z-type nucleic acids (Z-DNA/Z-RNA) (41). Accumulation of mitochondrial reactive oxygen species (ROS) promotes Z-RNA formation and aggregation in stress granules, which are recognized and bound by ZBP1 to activate subsequent programmed necroptosis (42). Meanwhile, nuclear stress signals further amplify damage sensing: DNA damage releases double-stranded DNA (dsDNA) fragments, which are recognized by AIM2 (43, 44); inactive telomeres transcribe long non-coding RNA TERRA, which is specifically captured by ZBP1 (45). Cellular senescence triggers the senescence-associated secretory phenotype (SASP), which includes multiple proinflammatory cytokines (e.g., IL-1β, TNF-α, IL-6), chemokines, and proteases (46).

### 3.2 Dynamic assembly of the PANoptosome complex

These trans-organellar damage signals ultimately converge on the dynamic assembly of the PANoptosome complex, primarily including the ZBP1-mediated, AIM2-mediated, and NLRP3-mediated PANoptosome complexes.

#### 3.2.1 ZBP1-mediated PANoptosome complex assembly

PANoptosis regulators such as ZBP1 and AIM2 are critical for sensing aging-related stress signals (e.g., mitochondrial DNA leakage and oxidative stress) (47, 48). Upon activation as a core sensor, ZBP1 relies on its receptor-interacting protein homotypic interaction motif (RHIM) domain. RIPK3 (receptor-interacting protein kinase 3) also contains an RHIM domain. ZBP1 recruits RIPK3 to the ZBP1-Z-RNA complex through homotypic RHIM-RHIM interactions between its own RHIM domain and that of RIPK3 (49). This interaction is based on specific binding between RHIM domains, allowing RIPK3 to stably associate with ZBP1 and become part of the complex.

FADD (Fas-associated death domain protein), an adaptor protein, plays a key role in apoptosis and necroptosis (50). Following RIPK3 recruitment, ZBP1 further recruits FADD to the complex through direct interactions (51). With RIPK3 and FADD successfully integrated into the ZBP1-Z-RNA complex, these molecules assemble into a ZBP1-Z-RNA-RIPK3-FADD multiprotein complex scaffold. This scaffold serves as the foundation for subsequent signal transduction and cell death events. Within this scaffold, RIPK3 kinase activity may be activated, phosphorylating downstream substrates such as MLKL (mixed lineage kinase domain-like protein) to initiate necroptotic signaling (52); concurrently, FADD recruits and activates caspase-8, triggering apoptotic pathways (53). In this manner, the initial ZBP1-Z-RNA-RIPK3-FADD scaffold becomes a critical starting point for cell death signaling, driving cells toward programmed death.

In the aged myocardium, ZBP1 exhibits cell-type-specific high expression primarily in cardiomyocytes and myocardial macrophages, whereas its expression is low in young myocardium and accumulates perinuclearly in cardiomyocytes with aging (54, 55). Immunofluorescence staining of aged myocardial tissue confirms the close colocalization of ZBP1 with the senescence marker p16INK4a and mitochondrial damage markers, indicating that ZBP1 activation is tightly coupled to cardiomyocyte senescence and mitochondrial dysfunction (54, 55). A key distinction between cardiac aging and acute injury lies in ZBP1's activation signal: in aging, ZBP1 is persistently activated by TERRA (telomere-derived RNA) from dysfunctional telomeres, driving sustained cardiomyocyte PANoptosis (45); in acute injury (e.g., myocardial ischemia-reperfusion), ZBP1 is transiently activated by viral Z-RNA or stress-induced endogenous Z-RNA (56). This cell-type-specific and signal-specific activation pattern underscores ZBP1's unique role in mediating age-related cardiac dysfunction.

#### 3.2.2 AIM2-mediated PANoptosome complex assembly

As a pattern recognition receptor, AIM2 specifically recognizes cytoplasmic double-stranded DNA (dsDNA), such as the mtDNA mentioned earlier (57). AIM2 contains a HIN-200 domain with high affinity for dsDNA, enabling direct binding to dsDNA (58). Upon binding to dsDNA, AIM2 undergoes a conformational change, transitioning from an inactive to an active state (59). Activated AIM2 engages in homotypic interactions between its N-terminal PYD (pyrin domain) and the PYD domain of the adaptor protein ASC (apoptosis-associated speck-like protein containing a CARD) (60, 61). ASC typically exists as a monomer in cells but polymerizes upon binding to activated AIM2. This polymerized ASC acts as a bridging molecule, connecting AIM2 to downstream effector molecules and facilitating its recruitment to the AIM2-dsDNA complex to form the AIM2-ASC complex (62).

The C-terminal CARD (caspase recruitment domain) of ASC interacts with the CARD domain of pro-caspase-1 (the inactive form of caspase-1) (63). Through this CARD-CARD interaction, ASC recruits pro-caspase-1 to the AIM2-ASC complex. Aggregation of multiple pro-caspase-1 molecules within the complex leads to mutual cleavage and activation, generating active caspase-1 and forming the AIM2 inflammasome (64), a key component of the AIM2-mediated PANoptosome complex. Beyond forming the core of the AIM2 inflammasome, the AIM2-ASC complex can recruit RHIM-containing proteins such as RIPK1 (receptor-interacting protein kinase 1) and RIPK3 under certain conditions (65). The incorporation of RIPK1 and RIPK3 expands the complex's functionality, enabling integration of necroptotic signaling pathways. Through the sequential recruitment and interaction of these molecules, the AIM2-mediated PANoptosome complex is ultimately formed.

In the aged myocardium, AIM2 exhibits distinct cell-type enrichment—predominantly expressed in cardiac fibroblasts and infiltrating macrophages, with minimal expression in cardiomyocytes, in contrast to the near-undetectable AIM2 levels in young cardiac fibroblasts (66, 67). This cell-specific expression aligns with AIM2's unique activation signal in aging: elevated mtDNA heteroplasmy in aged myocardium enhances mtDNA leakage into the cytoplasm, which directly activates AIM2 in fibroblasts to promote collagen secretion and myocardial fibrosis (68). In acute injury, by comparison, AIM2 is activated by dsDNA released from necrotic cells rather than age-related mtDNA leakage (66). This mechanism explains why AIM2 activation in aging is associated with chronic fibrotic remodeling, distinct from its role in acute tissue damage.

#### 3.2.3 NLRP3-mediated PANoptosome complex assembly

Both NLRP3 (NOD-like receptor protein 3) and AIM2 are pattern recognition receptors. NLRP3 can also form an inflammasome, ultimately generating a PANoptosome complex containing NLRP3, ASC, caspase-1, RIPK1, and RIPK3, which exhibits functions analogous to the AIM2-mediated PANoptosome complex.

In the aged myocardium, NLRP3 activation is cell-type-specific, primarily occurring in myocardial macrophages and vascular endothelial cells, with rare activation in cardiomyocytes (69, 70). A key factor in NLRP3 activation during aging is sustained mitochondrial ROS production—ROS levels in aged myocardium are significantly higher than in young myocardium, leading to chronic NLRP3 activation and persistent inflammatory signaling (29). This differs from acute injury, where NLRP3 is rapidly activated by ATP or potassium efflux (71, 72). The cell-type-specific and signal-specific activation of NLRP3 in aging mediates chronic myocardial inflammation, a hallmark of cardiac aging distinct from the acute inflammatory response to injury.

### 3.3 Downstream effects: cell death

The unique conformation of this multimodal complex enables it to simultaneously initiate three cell death pathways: apoptosis, necroptosis, and pyroptosis.

#### 3.3.1 Apoptosis

Upon activation, caspase-8 directly cleaves and activates downstream effector caspases (e.g., caspase-3, caspase-7), which in turn cleave intracellular substrates such as poly(ADP-ribose) polymerase (PARP), cytoskeletal components (actin, lamins), and other proteins. This leads to typical apoptotic morphological changes including nuclear condensation, chromosome agglutination, and membrane blebbing, ultimately resulting in cell apoptosis (73). Additionally, caspase-8 can cleave Bid to generate tBid, which translocates to mitochondria to promote the release of cytochrome c, thereby activating caspase-9 and triggering mitochondria-associated apoptotic events that culminate in cell death (74).

#### 3.3.2 Necroptosis

Activated RIPK3 phosphorylates MLKL, and phosphorylated MLKL translocates from the cytoplasm to the cell membrane, where it oligomerizes and inserts into the membrane to form pores. This causes membrane rupture, release of cellular contents, and induction of necroptosis (75). RIPK3 can also activate caspase-8 to exacerbate apoptosis (76).

#### 3.3.3 Pyroptosis

**Figure 2:**
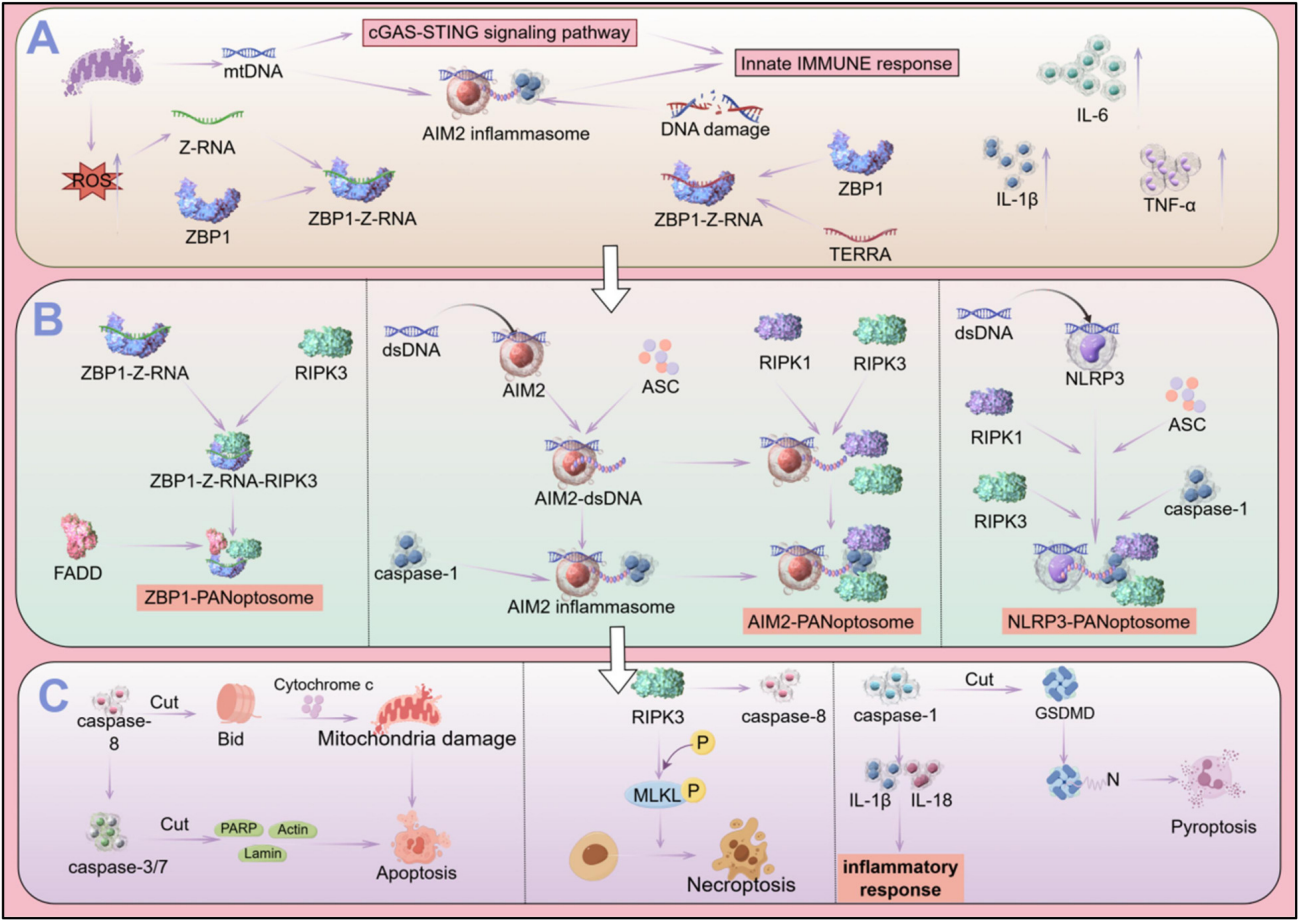
Molecular mechanisms and regulatory networks of PANoptosis. **(A)** Upstream damage sensing. In aging myocardium, mitochondrial dysfunction triggers signals: mtDNA leakage activates cGAS-STING and AIM2 inflammasome; mitochondrial ROS promotes Z-RNA aggregation, bound by ZBP1 to initiate necroptosis. Nuclear stress amplifies sensing: dsDNA activates AIM2; TERRA from inactive telomeres binds ZBP1; SASP releases pro-inflammatory factors for signal transduction. **(B)** PANoptosome assembly. Signals converge on complex assembly: ZBP1 recruits RIPK3 and FADD via RHIM; AIM2 recognizes dsDNA, forms inflammasome with ASC/caspase-1, and integrates RIPK1/3; NLRP3 forms a similar complex with analogous functions. **(C)** Downstream cell death. PANoptosome initiates apoptosis (caspase-8/-3/-7/-9 activation), necroptosis (MLKL phosphorylation/membrane pore formation), and pyroptosis (caspase-1-mediated IL-1β/IL-18 release and GSDMD pore formation).This figure was drawn by Figdraw.

Activated caspase-1 cleaves and activates pro-inflammatory cytokines IL-1β and IL-18, converting them into mature, biologically active cytokines that are released extracellularly to trigger inflammatory responses (77). Concurrently, caspase-1 cleaves Gasdermin D (GSDMD), releasing the N-terminal domain of GSDMD. This domain aggregates on the cell membrane to form pores, increasing membrane permeability and allowing efflux of ions and small molecules, influx of water, cellular swelling, and eventual rupture—hallmarks of pyroptosis (78) (Figure 2).

## 4 Key evidence for PANoptosis in cardiac aging and age-associated cardiovascular diseases

Cardiac aging-related processes involve two distinct but interconnected scenarios. Chronological cardiac aging refers to intrinsic time-dependent physiological deterioration of cardiac structure and function without overt pathological triggers (79). Age-associated cardiovascular diseases (CVDs) are pathological conditions where aging synergizes with external factors such as metabolic disorders, genetic mutations or ischemia to exacerbate cardiac damage (80). PANoptosis participates in both processes but with different activation mechanisms and causal contributions.

### 4.1 PANoptosis in chronological cardiac aging (physiological aging)

Chronological cardiac aging involves age-related degenerative changes in the hearts of healthy individuals or animal models. It is driven by intrinsic factors including mitochondrial dysfunction, telomere attrition and senescence-associated secretory phenotype (SASP) activation (81). Emerging preclinical evidence suggests that PANoptosis is closely associated with this physiological deterioration.

NLRP3 inflammasome contributes to age-related cardiac dysfunction. Aged NLRP3 knockout mice, a classic model of chronological aging, show prolonged lifespan and resistance to age-related myocardial injury. They also exhibit improvements in insulin sensitivity and IGF-1/leptin-adiponectin balance (69). This suggests NLRP3-mediated PANoptosis is associated with the progression of physiological cardiac aging, potentially through myocardial damage and metabolic imbalance. The myocardial protection observed in NLRP3 knockout mice may partially stem from improved systemic metabolism such as insulin sensitivity rather than direct inhibition of cardiac PANoptosis. However, direct evidence of NLRP3-PANoptosome assembly in the myocardium of aged wild-type mice is still lacking.

A mitochondrial damage-PANoptosis-inflammator*y* axis operates in healthy aging. In healthy humans, mtDNA heteroplasmy increases by 58.5% in individuals over 70 compared to those under 40 (68). This is accompanied by sustained elevation of serum IL-18, a key downstream effector of PANoptosis (82). Cardiac magnetic resonance studies in healthy elderly populations link myocardial fibrosis, a hallmark of physiological aging, to mitochondrial dysfunction (83). This implies mtDNA leakage, a trigger of AIM2/NLRP3 inflammasomes, may induce PANoptosis and be associated with age-related fibrosis. Direct detection of PANoptotic molecular markers such as GSDMD cleavage or RIPK3 phosphorylation in the myocardium of healthy elderly individuals has not been reported. The causal link between mtDNA heteroplasmy, PANoptosis activation and age-related fibrosis awaits verification through interventional studies.

ZBP1 may be involved in mediating cellular senescence in cardiac aging. Preclinical evidence suggests ZBP1 contributes to the acceleration of cellular senescence by upregulating expression of senescence markers p16INK4a and p21CIP1/WAF1 (54). LGR6 alleviates myocardial ischemia-reperfusion injury by inhibiting ZBP1 via the Wnt pathway (56). This points to ZBP1's potential role in age-related cardiac senescence. It is unclear whether ZBP1-mediated PANoptosis is specifically activated in cardiomyocytes during physiological aging as opposed to pathological stress. Whether ZBP1 knockout in aged mice can alleviate age-related cardiac functional decline independently of disease factors also requires confirmation.

### 4.2 PANoptosis in age-associated cardiovascular diseases

Age-associated CVDs including atrial fibrillation, coronary artery disease and diabetic cardiomyopathy arise from aging synergizing with external triggers. PANoptosis acts as a key mediator of disease progression in these contexts. Gut microbiota dysregulation promotes age-related atrial fibrillation. It upregulates atrial NLRP3 and its downstream effector caspase-1 in elderly individuals (70). This finding links NLRP3-mediated PANoptosis to the pathogenesis of age-related atrial fibrillation. NLRP3 activation in atrial tissue may be driven by age-related gut dysbiosis alone or by combined effects of aging and AF-related electrical remodeling. Whether PANoptosis inhibitors can specifically prevent AF recurrence in elderly patients compared to younger populations remains untested.

NLRP3 inhibitors exert therapeutic effects in age-related heart failure. Compounds such as MCC950 and OLT1177 alleviate cardiac fibrosis, hypertrophy and heart failure in Tet2-deficient mice while improving cardiac function (71, 72). Tet2 deficiency is associated with clonal hematopoiesis, an age-related condition increasing CVD risk (84), suggesting NLRP3-PANoptosis may mediate age-related heart failure progression. Tet2-deficient mice exhibit systemic inflammatory dysregulation though (85). The observed therapeutic effects may involve systemic inflammation suppression rather than direct cardiac PANoptosis inhibition. Further studies using cardiac-specific Tet2 knockout models are needed to confirm cardiac-intrinsic mechanisms.

AIM2 contributes to diabetic cardiomyopathy. In streptozotocin-induced diabetic rats, a model of age-related metabolic disorder, cardiac AIM2 expression is significantly elevated (66). *In vitro* experiments show high glucose treatment increases AIM2 levels in H9c2 cardiomyocytes (66). This implies high glucose, a key driver of diabetic cardiomyopathy, may induce the activation of AIM2-mediated PANoptosis. Uncertainties include the fact that high glucose, oxidative stress and advanced glycation end products may each independently drive PANoptosis. The specific contribution of age itself vs. diabetes severity remains unclear. Whether age-related declines in insulin sensitivity synergize with high glucose to amplify PANoptosis requires further investigation.

AIM2 and RIPK3 are linked to coronary artery disease and myocardial infarction. Serum AIM2 levels are significantly higher in CAD patients than in controls and AIM2 serves as an independent predictor of poor short-term prognosis (67). In acute MI patients, elevated plasma RIPK3 levels after PCI correlate with adverse outcomes. Recombinant RIPK3 exacerbates myocardial injury while RIPK3 antibodies exert protective effects (86). These findings link AIM2/RIPK3-mediated PANoptosis to CAD/MI progression. Whether targeting PANoptosis can improve long-term prognosis in elderly CAD/MI patients who often have multiple comorbidities remains unproven.

PANoptosis is involved in specific cardiomyopathies. In dilated cardiomyopathy, arrhythmogenic cardiomyopathy with DSP mutation and doxorubicin-induced cardiomyopathy, PANoptosis is characterized by co-activation of apoptosis marked by caspase-3/8 activation, necroptosis indicated by RIPK1/RIPK3/MLKL phosphorylation and pyroptosis shown by elevated GSDMD/ASC (87, 88). These data support the activation of PANoptosis in pathological cardiomyocyte death. Whether age-related PANoptosis primes the heart for doxorubicin-induced injury, a common concern in elderly cancer patients, requires age-stratified studies.

JAK2V617F-related clonal hematopoiesis links AIM2 to cardiovascular diseases. Human monocyte/macrophage THP-1 cells expressing JAK2V617F show increased AIM2 expression and higher levels of the AIM2 inflammasome (89). JAK2-CH is closely associated with age-related cardiovascular diseases, suggesting a potential role of AIM2-mediated PANoptosis in disease progression.

The mitochondrial damage-inflammation-fibrosis axis may form a potential positive feedback loop in age-associated CVDs. Metabolic disorders such as diabetes and genetic mutations such as JAK2V617F amplify mitochondrial damage and death signals by upregulating AIM2/NLRP3 inflammasomes. This further exacerbates oxidative stress and the inflammatory microenvironment, promoting cardiac fibrosis and dysfunction (83).

### 4.3 Operational definition and evidence tier criteria of PANoptosis in cardiac aging

To distinguish PANoptosis from the mere co-activation of apoptosis/pyroptosis/necroptosis markers, we define operational criteria for PANoptosis in cardiac aging: A lytic programmed cell death process mediated by the PANoptosome complex (integrating core molecules of apoptosis, pyroptosis, and necroptosis), which simultaneously activates at least two of the three death pathways and directly contributes to cardiac structural remodeling or functional deterioration.

Evidence tier classification is established based on the rigor of experimental validation to standardize the evaluation of PANoptosis-related studies.The detailed criteria for each evidence tier are summarized in Table 1.

**Table 1:** Evidence tier criteria for PANoptosis in cardiac aging.

Evidence tier	Criteria description
Strong	Direct detection of PANoptosome assembly (e.g., co-immunoprecipitation of key components including ZBP1/RIPK3/ASC/caspase family members) + simultaneous activation of ≥2 death pathways (apoptosis/pyroptosis/necroptosis) + PANoptosome-specific intervention (e.g., complex disruption, core component knockout) alleviates cardiac aging-related phenotypes (e.g., hypertrophy, fibrosis, dysfunction).
Moderate	Simultaneous activation of ≥2 death pathways (e.g., caspase-3/8 for apoptosis, GSDMD/IL-1β for pyroptosis, RIPK3/MLKL for necroptosis) + upregulation of PANoptosis key regulators (ZBP1/AIM2/NLRP3) + intervention targeting these regulators (e.g., inhibitor treatment, gene silencing) alleviates cardiac aging-related damage.
Weak	Activation of a single death pathway + indirect association with PANoptosis regulators (e.g., upregulation of NLRP3 without pathway crosstalk) + no direct link to cardiac aging phenotypes or lack of intervention validation.

The evidence summarized in Table 2 systematically classifies the aforementioned studies into distinct tiers based on standardized criteria, further validating that PANoptosis-driven cardiac aging is a synergistic cascade rather than isolated activation of individual death pathways. Strong and moderate preclinical evidence collectively indicate that the NLRP3-AIM2-ZBP1-RIPK3 signaling network is closely linked to cardiac aging, while weak evidence highlights critical directions for future research (e.g., direct PANoptosome detection in clinical samples, validation of pathway crosstalk). Based on this rigorously classified evidence, targeted intervention strategies against PANoptosis have become a promising approach to delay cardiac aging, which is elaborated in the following section.

**Table 2:** Summary of studies on the relationship between PANoptosis and cardiac aging under different evidence levels.

Evidence tier	Study model	Core findings	Remarks	Reference
Strong	Doxorubicin-induced cardiomyopathy (mouse) + H9c2 cells	FUNDC1 deficiency promotes PANoptosome formation (TUFM-mtDNA interaction disruption) + co-activation of caspase-3/8 (apoptosis), GSDMD (pyroptosis), and RIPK3/MLKL (necroptosis); FUNDC1 overexpression alleviates cardiac dysfunction	Directly demonstrates PANoptosome-mediated multi-pathway activation and functional rescue	(87)
Moderate	Desmoplakin cardiomyopathy (DSP mutation, human/mouse)	Co-activation of caspase-3/8 (apoptosis), GSDMD/ASC (pyroptosis), and RIPK1/RIPK3/MLKL (necroptosis); ZBP1/AIM2/NLRP3 upregulation	Confirms multi-pathway co-activation and key regulator elevation, lacks direct PANoptosome detection	(88)
Moderate	NLRP3 knockout (aged male mice)	NLRP3 deletion reduces caspase-1 activation (pyroptosis) and caspase-3 cleavage (apoptosis), improves cardiac hypertrophy/fibrosis and longevity	Validates NLRP3-mediated cross-talk between two death pathways and therapeutic benefit	(69)
Moderate	Tet2-deficient cardiomyopathy (mouse)	NLRP3/IL-1β axis activation (pyroptosis) + caspase-3 upregulation (apoptosis); NLRP3 inhibitors (MCC950) alleviate cardiac failure	Links NLRP3 to dual pathway activation and responsive cardiac dysfunction	(71)
Moderate	Type 2 diabetic cardiomyopathy (rat) + high glucose-treated H9c2 cells	AIM2 upregulation + caspase-1 activation (pyroptosis) and caspase-3 activation (apoptosis); AIM2 silencing alleviates myocardial injury	Demonstrates AIM2-dependent dual pathway activation and protective effect of targeting AIM2	(66)
Weak	Cellular senescence model (human cardiomyocytes)	ZBP1 upregulation promotes p16INK4a/p21CIP1/WAF1 expression + caspase-8 activation (apoptosis)	Only single death pathway activation linked to PANoptosome regulator (ZBP1), no cardiac aging phenotype rescue	(54)
Weak	Coronary artery disease (human, *n* = 279)	Serum AIM2 elevation correlates with poor short-term prognosis; no direct detection of death pathways	Indirect association of PANoptosome regulator (AIM2) with disease prognosis, lacks pathway activation evidence	(67)
Weak	Aged population (human, ≥60 years)	IL-18 elevation (pyroptosis marker) correlates with physical dysfunction; no PANoptosome regulator data	Only single pyroptosis marker association with aging-related dysfunction, no link to PANoptosis core molecules	(82)

## 5 Targeting PANoptosis: intervention strategies for anti-cardiac aging

### 5.1 Small molecule inhibitors

#### 5.1.1 Inhibition of the ZBP1-RIPK3 axis

As a critical sensor of innate immunity, ZBP1 activates PANoptosis by recognizing viral Z-RNA or endogenous nucleic acids (90). Among them, the Z*α*2 domain of ZBP1 plays a key role in the PANoptosis signaling pathway. studies using a ZBP1 mouse model with a deleted Z*α*2 domain (Zbp1*Δ*Z*α*2) have demonstrated that this domain is essential for influenza A virus-induced PANoptosis and perinatal lethality (91). Developing small molecule inhibitors that specifically target this domain to suppress ZBP1 activity and block PANoptosis initiation holds preclinical promise as a potential therapeutic approach for cardiac aging.

RIPK3 is one of the core death-executing molecules in PANoptosis, and regulating its activity is crucial for intervening in cardiac aging. Allosteric inhibitors of RIPK3 bind to RIPK3 to alter its conformation and inhibit its kinase activity. For example, the RIPK1 inhibitor GSK547 significantly reduces atherosclerotic lesion area, inhibits inflammatory cytokines (MCP-1, IL-1β, TNF-α), and monocyte infiltration in the early stage (2 weeks) (92). In 2013, Kaiser et al. first identified three classes of small molecule RIPK3 inhibitors (GSK840, GSK843, and GSK872), which exhibit good RIPK3 inhibitory activity but suffer from high cytotoxicity, poor drug-likeness, and induction of RIPK3-dependent apoptosis (93, 94). In 2018, Park et al. discovered the RIPK3 kinase inhibitor HS1371, which has an IC50 value of 20.8 nM for RIPK3 inhibition, effectively suppresses necroptosis in HT29 and L929 cells without causing apoptosis, though *in vivo* data remain unreported (95). Developing highly selective and specific RIPK3 inhibitors may reduce cardiac cell death and delay cardiac aging.

#### 5.1.2 Blocking PANoptosome complex assembly

Compounds and endogenous molecules targeting PANoptosome formation-related molecules represent promising therapeutic strategies (96). Current studies have shown that peptide inhibitors can interfere with protein-protein interactions; for example, nanobodies targeting FADD block the formation of death signal complexes (90). ASC plays a key role in PANoptosome assembly as a critical bridge connecting ZBP1 and effector molecules (e.g., RIPK3, RIPK1), and Sundaram B et al. found that ASC deficiency significantly inhibits PANoptosome formation (97). Another study showed that baicalin inhibits PANoptosis in macrophages by blocking mitochondrial Z-DNA formation and ZBP1-PANoptosome assembly, exerting protective effects against inflammatory diseases (98). Future development of related formulations may further block death signal complex formation and optimize therapeutic effects against cardiac aging.

### 5.2 Gene editing and gene therapy

#### 5.2.1 Myocardial-specific ZBP1 knockout

Myocardial-specific ZBP1 knockout can specifically inhibit the PANoptosis signaling pathway in the heart without affecting other tissues. CRISPR-Cas9 technology, a powerful gene editing tool capable of precise knockout of specific genes, was first reported in 2012 for its programmable site-specific DNA cleavage ability. *In vitro* studies showed that the CRISPR system using Cas9 can cleave any DNA strand, laying the foundation for the field's development (99), which has now been applied to cardiac disease research. For example, knocking down Nr1d1 via CRISPR/Cas9 significantly inhibits cardiac cell senescence, promotes proliferation, and reduces apoptosis (100). In a rat model of myocardial ischemia-reperfusion injury (MIRI), adenovirus-mediated ZBP1 overexpression significantly exacerbates myocardial PANoptosis, while CRISPR/Cas9-mediated ZBP1 knockout alleviates this effect (55). This precision gene editing-based regulatory approach opens new research directions for targeted intervention in cardiac aging.

#### 5.2.2 siRNA targeting RIPK3

RNA interference (RNAi) is a method for specifically inhibiting gene expression via small interfering RNA (siRNA). Designing and synthesizing siRNA targeting RIPK3 may potentially suppress RIPK3 expression and block PANoptosis initiation. This approach offers advantages of high efficiency, strong specificity, and ease of operation, providing a convenient pathway for regulating genes associated with cardiac aging.

### 5.3 Combination therapy strategies

Monotherapies targeting a single pathway may have limited efficacy, whereas combination strategies can exert synergistic effects to enhance therapeutic outcomes. Senolytics (e.g., dasatinib + quercetin), which selectively eliminate senescent cells, may synergistically inhibit cardiac cell death and clear senescent cells when combined with PANoptosis inhibitors to combat cardiac aging. However, no current studies have confirmed this mechanism.

### 5.4 Novel delivery systems to enhance targeting

#### 5.4.1 Cardiac-targeted nanoparticles

As a novel drug delivery system, nanoparticles exhibit high targeting ability and biocompatibility. Encapsulating RIPK1 inhibitors in liposomes enables cardiac-targeted delivery, enhancing efficacy while reducing side effects. For example, liposome-encapsulated RIPK1 inhibitors (e.g., RGD/PEG-modified liposomes) enhance drug accumulation by targeting myocardial integrin αvβ3 (101). Development of such intelligent delivery systems marks a significant step toward precision-targeted cardiac drug therapy.

#### 5.4.2 Exosomal delivery

**Figure 3:**
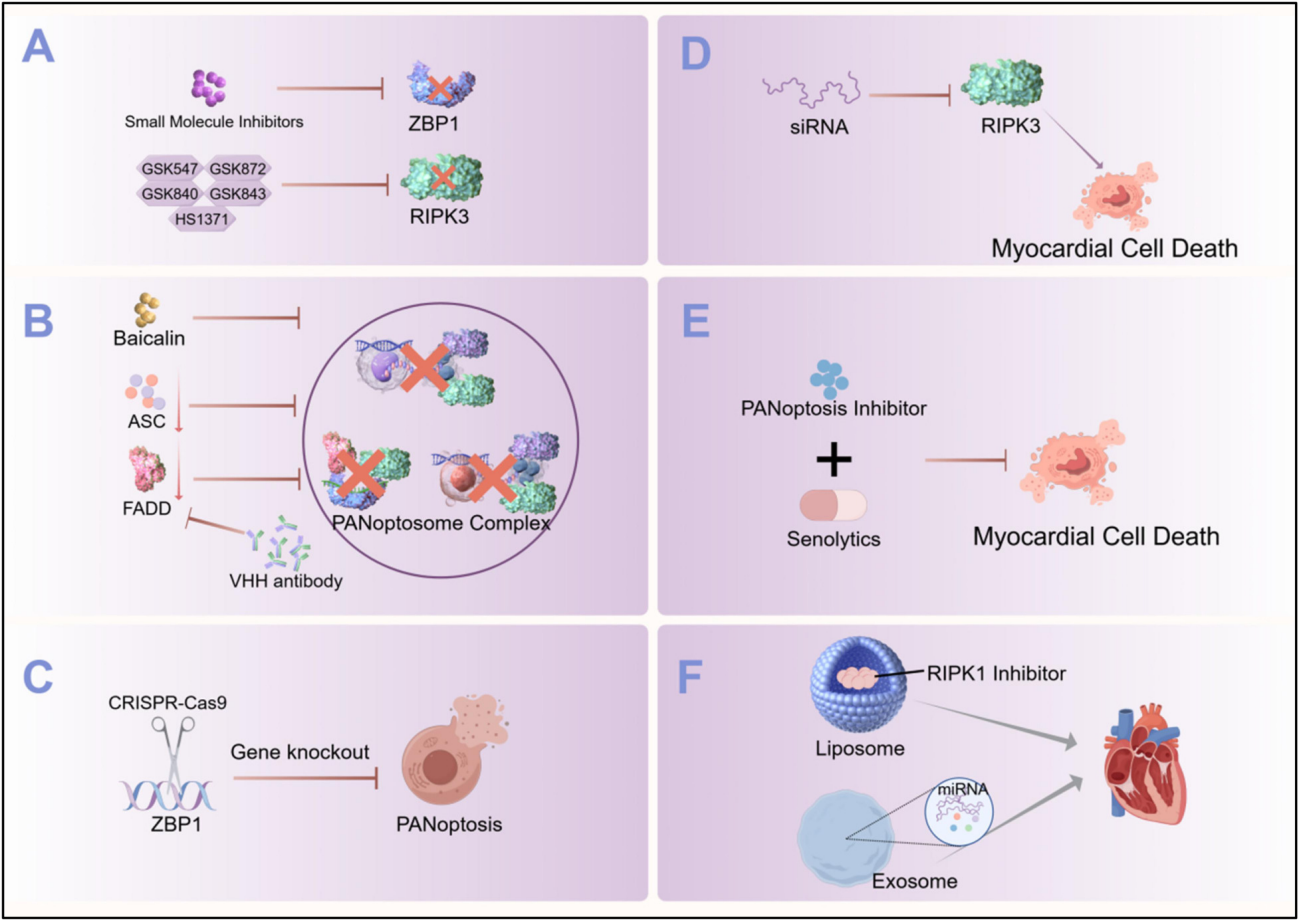
Targeting PANoptosis: an interventional strategy against cardiac aging. **(A)** Small molecule inhibitors targeting the Zα2 domain of ZBP1 or RIPK3 kinase activity to block PANoptosis initiation. **(B)** Blockade of PANoptosome assembly using compounds (e.g., Baicalin) or biomolecules (e.g., VHH antibody against FADD, ASC-targeting agents) to disrupt complex formation and inhibit PANoptosis. **(C)** Gene knockout of ZBP1 to specifically abrogate PANoptosis signaling in cardiomyocytes. **(D)** siRNA-mediated knockdown of RIPK3 to suppress expression and prevent myocardial cell death. **(E)** Combination therapy utilizing PANoptosis inhibitors with senolytics to synergistically inhibit cell death and clear senescent cells. **(F)** Novel delivery systems (e.g., ligand-modified liposomes, exosomes) for targeted delivery of therapeutics (e.g., RIPK1 inhibitors) to the heart, enhancing efficacy and reducing off-target effects.This figure was drawn by Figdraw.

Exosomes, nanoscale vesicles secreted by cells, possess natural targeting ability and biocompatibility, capable of penetrating biological barriers (e.g., blood-brain barrier, placental barrier) and being recognized and internalized by specific receptor cells (102, 103). They can carry various drug molecules (e.g., nucleic acids, proteins, lipids), improving therapeutic effects through targeted delivery while minimizing side effects (104, 105). Studies on exosome applications in cardiac repair have shown that they can improve cardiac function by delivering drug molecules (e.g., miRNAs or small molecules) (106). This breakthrough application of natural carrier technology opens a frontier for cardiac repair therapy based on bioactive delivery (Figure 3).

### 5.5 Challenges in clinical translation

#### 5.5.1 Tissue specificity and safety

In clinical applications, PANoptosis-targeted drugs may exhibit off-target effects, i.e., adverse impacts on non-target tissues. Studies have shown that RIPK3 inhibitors may interfere with immune homeostasis (107). To minimize off-target effects, the development of highly tissue-specific drug molecules and delivery systems is required. For example, myocardial-specific promoters or antibodies can achieve cardiac tissue-specific targeting, reducing adverse effects on other tissues.

Some PANoptosis-targeted drugs may suppress immune function, increasing risks of infection and tumorigenesis. Previous studies have indicated that long-term inhibition of the ZBP1-RIPK3 axis may elevate infection risks (108). To mitigate immunosuppressive risks, the impact on the immune system must be fully considered during drug design and clinical application, with corresponding measures taken to protect immune function—such as combining with immunomodulators or developing drug molecules with immunomodulatory functions.

#### 5.5.2 Individual heterogeneity

Genetic background variations among individuals may affect the efficacy and safety of PANoptosis-targeted drugs. To improve drug efficacy and safety, individual genetic differences must be fully considered to enable personalized therapy. For example, genetic testing and analysis can inform selection of appropriate drugs and treatment regimens based on individual genetic profiles.

#### 5.5.3 Intervention time window

Cardiac aging is a progressive process, and early intervention is critical for delaying its progression. PANoptosis-targeted drugs need to be administered at the early stage of cardiac aging to achieve optimal efficacy. Therefore, the establishment of effective early diagnostic methods and intervention strategies is essential to timely detect signs of cardiac aging and initiate corresponding interventions.

## 6 Summary and prospects

Cardiac aging is a complex multifactorial process involving interactions of multiple cellular and molecular mechanisms. In recent years, PANoptosis, as an integrative cell death modality, has gradually emerged as a new focus in cardiac aging research. Its unique molecular mechanism—forming a dynamically regulated “death signaling network” by integrating key pathways of apoptosis, pyroptosis, and necroptosis—provides a new perspective for understanding the pathophysiological mechanisms of cardiac aging. Preclinical studies suggest that PANoptosis is closely associated with cardiac aging, potentially contributing to the decline of cardiac function by accelerating myocardial cell loss, fibrosis, and chronic inflammation. Based on this mechanism, PANoptosis-targeted intervention strategies (such as gene editing, RNAi, combination therapy, and novel delivery systems) have demonstrated enormous therapeutic potential, offering new directions for delaying cardiac aging. This review provides a theoretical foundation and practical guidance for developing PANoptosis-targeted anti-cardiac aging strategies, holding promise to make significant contributions to achieving healthy aging.

Furthermore, the discussion of cardiac aging must also consider the impact of modern cardiovascular therapeutics, particularly cardiac implantable electronic devices (CIEDs), which are increasingly used in an aging population. While CIEDs such as pacemakers, implantable cardioverter-defibrillators (ICDs), and cardiac resynchronization therapy (CRT) devices significantly prolong life and improve quality of life, their long-term presence may inadvertently influence aging pathways. Chronic device-related inflammatory responses, mechanical stress, and electrical remodeling could potentially modulate microenvironmental signals that affect PANoptosis activation, fibrosis, and cellular senescence. This underscores the need to evaluate not only the direct benefits of such devices but also their long-term biological interactions with cardiac aging processes (109). Concurrently, as CIED utilization rises in the elderly, the psychological and emotional dimensions of device acceptance become crucial. Younger patients often report greater device-related anxieties, limitations in daily and professional life, and unmet informational needs, whereas older patients may experience improved quality of life post-implantation. Psychological stress and poor adaptation can activate neuroendocrine and inflammatory pathways, which may in turn exacerbate cardiac aging. Thus, integrating psychological assessment and support into the management of elderly patients with CIEDs is essential, as it may help mitigate stress-induced acceleration of cardiac decline and improve overall treatment outcomes (110). To provide a systematic overview of the clinical translation potential discussed throughout this review, we summarize the key intervention strategies, their target conditions, and expected outcomes in Table 3.

**Table 3:** Clinical perspectives on intervention strategies for delaying cardiac aging.

Clinical perspective	Diseases/conditions potentially delayed or avoided	Key intervention strategies	Expected outcome
PANoptosis-targeted therapy	Heart failure, myocardial fibrosis, arrhythmias	ZBP1/RIPK3 inhibitors, gene editing, siRNA	Reduced cardiomyocyte death, slowed structural remodeling
Senolytic combination therapy	Senescence-associated cardiomyopathy, diastolic dysfunction	Senolytics (e.g., dasatinib + quercetin) + PANoptosis inhibitors	Synergistic clearance of senescent cells, dampened inflammation
Integrated psychological intervention	Psychogenic arrhythmias, anxiety/depression-related decline	Psychological support, cognitive behavioral therapy, device education	Improved psychological adaptation, reduced sympathetic activation
Personalized device management	Device-related complications, increased psychological burden	Remote monitoring, individualized programming, end-of-life device care planning	Enhanced quality of life, reduced unnecessary device interventions
Early diagnosis and prevention	Subclinical cardiac aging, early diastolic dysfunction	Biomarker screening, imaging assessment, genetic risk evaluation	Early intervention, delayed disease progression

However, despite progress, the mechanisms of PANoptosis in cardiac aging and its clinical application still face numerous challenges. First, the molecular regulatory network of PANoptosis has not been fully elucidated, particularly its dynamic changes across different aging stages and pathological conditions, which require further exploration. Second, PANoptosis-targeted intervention strategies in clinical translation face issues such as tissue specificity, safety, and individual heterogeneity. For example, key unresolved questions include how to reduce off-target effects and immunosuppressive risks, how to design personalized treatment regimens based on individual genetic backgrounds, and how to determine the optimal intervention time window. Additionally, while the development and application of novel delivery systems (such as nanoparticles and exosomes) are promising, their large-scale production and clinical validation still require further optimization.

Looking to the future, research in this field needs to break through three dimensions: at the mechanistic level, multi-omics technologies and single-cell analysis should be used to comprehensively reveal the death signature profiles of different cell subsets in aging hearts and decipher the cross-cell communication mechanisms mediated by PANoptosis among endothelial cells, fibroblasts, and immune cells; in technological development, efforts should focus on overcoming the efficiency bottleneck of cardiac-targeted drug delivery and developing intelligent nanocarriers with mechanical response properties to adapt to the stiffened microenvironment of aging myocardium; it is essential to develop more tissue-specific and safe drug molecules and delivery systems while exploring the synergistic effects of combination therapy to enhance efficacy and reduce side effects. In clinical translation, there is an urgent need to establish personalized treatment stratification criteria based on the degree of PANoptosis activation, predict patient-specific drug sensitivity through organ-on-a-chip technologies, establish early diagnostic markers and intervention time windows for cardiac aging, and conduct large-scale clinical trials to validate the efficacy and safety of PANoptosis-targeted therapeutic strategies.

It is anticipated that the interdisciplinary integration of synthetic biology and artificial intelligence may give rise to the design of gene circuits capable of dynamically regulating cell death pathways, providing potential revolutionary tools for alleviating cardiac aging. Through multidisciplinary collaborative innovation, PANoptosis-targeted intervention strategies are poised to become a critical breakthrough for achieving healthy aging.
